# Mitochondrial DNA editing: Key to the treatment of neurodegenerative diseases

**DOI:** 10.1016/j.gendis.2024.101437

**Published:** 2024-09-21

**Authors:** Ye Hong, Ying Song, Wenjun Wang, Jinghui Shi, Xi Chen

**Affiliations:** a Department of Pharmacology, Zhejiang University of Technology, Hangzhou, Zhejiang 310014, China; b Hangzhou King's Bio-pharmaceutical Technology Co., Ltd., Hangzhou, Zhejiang 310007, China

**Keywords:** Base editor, CRISPR-Cas9, Mitochondrial DNA, mitoTALENs, mitoZFNs, Neurodegenerative diseases

## Abstract

Neuronal death is associated with mitochondrial dysfunction caused by mutations in mitochondrial DNA. Mitochondrial DNA becomes damaged when processes such as replication, repair, and nucleotide synthesis are compromised. This extensive accumulation of damaged mitochondrial DNA subsequently disrupts the normal function of mitochondria, leading to aging, degeneration, or even death of neurons. Mitochondrial dysfunction stands as a pivotal factor in the development of neurodegenerative diseases, including Parkinson's disease, Alzheimer's disease, Huntington's disease, and amyotrophic lateral sclerosis. Recognizing the intricate nature of their pathogenesis, there is an urgent need for more effective therapeutic interventions. In recent years, mitochondrial DNA editing tools such as zinc finger nucleases, double-stranded DNA deaminase toxin A-derived cytosine base editors, and transcription activator-like effector ligand deaminases have emerged. Their emergence will revolutionize the research and treatment of mitochondrial diseases. In this review, we summarize the advancements in mitochondrial base editing technology and anticipate its utilization in neurodegenerative diseases, offering insights that may inform preventive strategies and therapeutic interventions for disease phenotypes.

## Introduction

Mitochondria are essential organelles for the normal functioning of eukaryotic cells and play a key role in cellular activities including apoptosis, calcium homeostasis, signal transduction, and regulation of reactive oxygen species levels.^1^ The endosymbiotic theory proposes that mitochondria originated from aerobic bacteria phagocytized by primitive eukaryotic organisms, forming a symbiotic relationship with the host cell.^2^ Most of the DNA of the original bacterial endosymbiont has been lost or transferred to the nucleus, leaving a much smaller circular molecule, now known as mitochondrial DNA (mtDNA).^3^ While distinct from the nuclear genome, mtDNA interacts with it and is indispensable for maintaining mitochondrial function.^4^ Upon exposure to external or internal stimuli, mitochondria undergo perturbations in crucial processes, including mtDNA replication, repair, and nucleotide synthesis, ultimately disrupting mtDNA homeostasis. This disruption significantly impacts neurons, characterized by their high-energy demands and limited regenerative capabilities.^5^ Recent studies have emphasized the critical role of mitochondria in the pathogenesis of neurodegenerative diseases (NDDs), particularly the impact of mtDNA mutations on the development of Parkinson's disease (PD) and Alzheimer's disease (AD).^6^

Given the significance of mtDNA mutations in numerous diseases, researchers have sought ways to mimic or repair these alterations. However, the intricate double membrane structure of mitochondria poses a formidable obstacle for traditional DNA editing tools, particularly CRISPR-based systems.^7^ To overcome this limitation, mitochondrial gene editing technologies have emerged as promising alternatives. Nuclease-based mtDNA editors, such as mitoZFN and mitoTALEN, have demonstrated the ability to penetrate mitochondria and perform targeted double-stranded cleavage of mutant mtDNA molecules. While these tools effectively alter the mutant/wild-type mtDNA ratio, they are limited in their capacity to mimic or repair specific mutations.^8^ In contrast, mitochondrial base editing technologies (*e.g.*, DdCBE) can specifically target the repair of mutated mtDNA and drive the transition from the heterogeneous state to a healthy wild-type mtDNA population.^9^ Based on these advancements, we hypothesized that mitochondrial base editing technology may provide a new direction for the prevention or treatment of NDDs. In this review, we discuss recent advances in mitochondrial gene editing technology and explore the relationship between mtDNA mutations and four common NDDs, aiming to inform the prevention of NDDs or the treatment of disease phenotypes.

## Mitochondrial genome and mutations

Mitochondria contain a separate genome, and the full sequence of the mitochondrial genome was determined by Anderson et al in 1981.^10^ Human mtDNA is a double-stranded circular DNA (dsDNA) molecule containing 16,569 bp with light (L) and heavy (H) strands, each of which can be transcribed. mtDNA is intronless and polycistronic, and its genes encode 22 tRNAs, 2 rRNAs, and 13 polypeptides that are components of the electron transport chain.^11^

**Figure 1:**
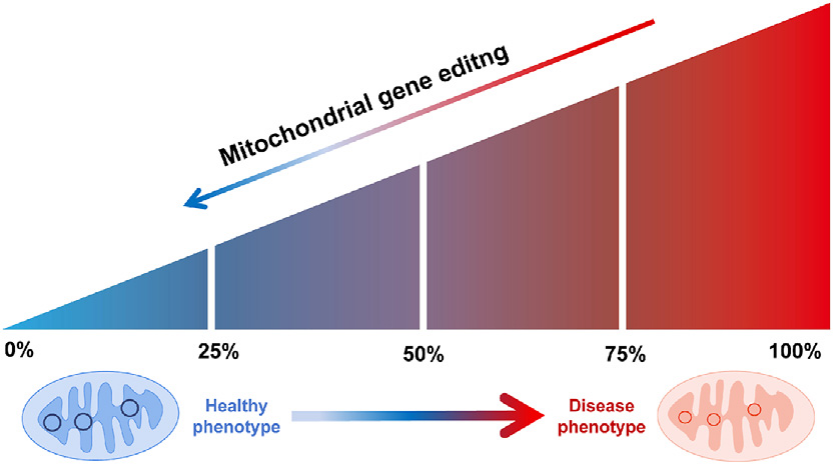
Manipulating “thresholds” with mitochondrial gene editing tools. Within heterogeneous mitochondria, the ratio of mutations to wild type dictates the initiation of phenotypic expression. Once the mitochondrial DNA mutation surpasses a certain threshold, the disease phenotype manifests. By employing mitochondrial DNA gene editing techniques, the degree of heteroplasmy can be adjusted to lower the ratio of mitochondrial DNA mutations below the disease threshold.

mtDNA differs from nuclear DNA in that it does not follow the Mendelian laws of inheritance, since mitochondria in a fertilized egg originate almost exclusively from the mother. Consequently, mtDNA is maternally transmitted to offspring.^12^ In addition, mtDNA exhibits a higher mutation rate than nuclear DNA due to several factors. Firstly, its proximity to the inner mitochondrial membrane exposes it to potentially damaging conditions. Secondly, the lack of protection from histones and DNA-binding proteins makes it highly susceptible to free-radical attack. Lastly, mtDNA lacks an efficient repair system and protective mechanisms to counteract these threats, further contributing to its increased mutation rate. Therefore, mtDNA is prone to mutations.^13^ Heterogeneity is present in mitochondria when both mutant and wild-type sequences coexist. In such heterogeneous mitochondria, a damaged phenotype manifests only when a specific proportion of mutant mtDNA is surpassed; this is known as the “threshold effect".^14^ Thus it is only necessary to reduce the proportion of mutated mtDNA below the disease threshold to restore the clinical phenotype, a property that gene therapy exploits (Fig. 1).^15^

If the mtDNA mutation reaches a certain threshold, mitochondrial function becomes abnormal, and oxidative phosphorylation becomes impaired. This blocks cellular energy production and causes the cell to lose viability due to an insufficient energy supply, which may lead to organismal senescence. Recent studies have shown that the increase in aging-associated mtDNA mutations is not caused by the accumulation of damage but by the clonal amplification of mtDNA replication errors that occur during development.^16^ Lokesh et al knocked down the mtDNA polymerase γ (PolgA D257A) in mice, causing the mice to accumulate mtDNA mutations as they aged. They observed that the mice developed mitochondrial bioenergetic defects, amyloid accumulation, and brain atrophy.^17^ The causal relationship between mtDNA and aging remains unclear, however, there is increasing evidence that somatic mtDNA mutations are elevated in age-related disorders such as AD, PD, and Huntington's disease (HD), particularly in the brain tissue of affected individuals.^18^

## Mitochondrial gene editing

In recent years, gene therapy technology has developed rapidly, particularly the CRISPR/Cas9 system, which can conveniently cleave DNA in the presence of guide RNA (gRNA).^19^ However, its application in mtDNA editing is still controversial, mainly for the following reasons: (i) the mitochondrial genome is relatively small and lacks sufficient CRISPR-editable sites; (ii) CRISPR relies on gRNAs for effectiveness, but there is no efficient method to introduce gRNAs into mitochondria.^20^ Currently, mitochondrial gene editing techniques are based on two approaches: nuclease-based approaches and base editing approaches. We provide a detailed review of mitochondrial gene editing technologies and discuss their advantages and disadvantages (Table 1). Because effective delivery methods are critical to the success of mitochondrial gene editing technologies, we also focused on the current developments and challenges in vector delivery strategies.

**Table 1:** Summary of the main mtDNA editing tools.

	mtZFN	mitoTALEN	CRISPR-Cas9	DdCBE	mitoZFDs	TALEDs	mitoBEs
Major component	ZF + Fok I + MTS	TALE + FokI + MTS	Cas9+MTS + sgRNA	TALE + DddA_tox_ + UGI + MTS	ZF + DddA_tox_ + UGI	TALE + DddA+ TadA8e + MTS	TALE + nickase + deaminase + UGI
Edit element	Fok I	Fok I	Cas9	Split-DddA_tox_ half	Split-DddA_tox_ half	Split-DddA_tox_ half	Deaminase
Editing type	Depletion	Depletion	Depletion	C > T	C > T	C > T and A > G	C > T and A > G
Off-target editing	High	High	High	High	High	Low	Low
Advantages	Small	High flexibility; easy to design	High specificity; easy operation and short cycle	Small; high specificity	Small; high specificity; unique mutation patterns	Small; high specificity	High efficiency; chain selectivity
Disadvantages	Difficult to design; limited targeting scope; time-consuming; reducing mtDNA copy number	Large; limited targeting scope; reducing mtDNA copy number	Limited targeting scope; PAM dependent	Only C > T conversion	Limited targeting scope; complexity	Bystander editing	Long-term effects and safety unknown

## Mitochondrial gene editing tools

### Artificial nuclease editing system

Artificial nuclease-mediated mitochondrial gene modification technologies (*e.g.*, mitoZFN and mitoTALEN) are highly programmable, specific, and flexible.^21^^,^^22^ ZFNs comprise zinc-finger proteins and the restriction endonuclease FokI. The zinc-finger protein consists of 3–6 ZF modules, with each ZF module recognizing and binding to a 3-bp DNA sequence.^23^ Transcription-activator-like effectors nucleases (TALEN) are gene editing tools derived from plant pathogenic bacteria. TALE is obtained from the bacterium, xanthomonas, with amino acid sequences corresponding to nucleotide sequences on the target DNA.^24^ TALE consists of four modules, and one modular unit recognizes one base, which is the key site for specific recognition of DNA.^25^ TALEN and ZFN are pure protein systems that can achieve targeted cleavage of mtDNA by adding mitochondria target sequence (MTS).^21^^,^^26^ It has been demonstrated in a mouse model carrying a pathogenic mutation in mtDNA (m.5024C > T) that the delivery of mitoZFN or mitoTALEN using AAV9.45 effectively reduces the level of mutant mtDNA and increases the wild-type mtDNA in various tissues such as heart and skeletal muscle.^26^^,^^27^ The effectiveness of artificial nuclease editing systems in targeted editing of mtDNA has been preliminarily demonstrated in *in vitro* and *in vivo* studies.

Artificial nuclease editing systems can specifically target and remove mutant mtDNA from heterogeneous cells and induce changes in mtDNA heterogeneity. mitoZFN has a rather low editing efficiency, and an iterative editing strategy requires a long time. Moreover, each of the zinc-finger protein groups binds to 3 bp of DNA, which may cause potential inaccuracies. TALE can recognize individual bases and thus has more flexible site selectivity, however, its arrays are more complicated to analyze and construct and need to be constructed from scratch every time the sites are replaced, which places a great burden on researchers and even makes it difficult to obtain effective structures.^28^ Therefore, the exploration and development of safer and more efficient gene delivery vectors is crucial for advancing gene therapy research targeting mtDNA. The emergence of such novel vectors is expected to significantly enhance the precision, efficiency, and safety of gene editing, thereby opening new possibilities for gene therapy for mtDNA-related diseases.

### CRISPR-Cas9

The CRISPR-Cas9 system is a widely used genome editing tool.^29^ The core mechanism lies in the ability to precisely induce double-strand breaks in specific target genomic regions, and realize the editing and modification of the target genome through intracellular DNA repair mechanisms, such as non-homologous end-joining, microhomology-mediated end-joining, and homologous recombination.^30^ The CRISPR-Cas9 system has effectively overcome the limitations existing in traditional technologies such as TALEN and ZFN. This is due to its user-friendliness and high degree of flexibility, opening up new paths for research and application in the field of gene editing.^31^ The successful application of the CRISPR/Cas9 system for mitochondrial gene editing was first reported in 2015, demonstrating its feasibility for mtDNA editing in mammalian mitochondria.^32^ To further explore its function and application in mitochondria, the researchers have modified and optimized the CRISPR system to achieve its efficient translocation to mitochondria and precise manipulation of mtDNA.^33^ Bian et al showed specific targeting of mtDNA by the mito CRISPR/Cas9 system, leading to the reduction of mtDNA copy number in human cells and zebrafish.^34^ Bi et al developed the mito-Cas9 system by adding mitochondrial targeting sequences and 3′ untranslated regions of nuclear-encoded mitochondrial genes upstream and downstream of Cas9 genes, achieving effective degradation of mtDNA, which provided direct evidence to demonstrate the feasibility of the CRISPR-Cas9 system in mtDNA editing.^35^

There is still a heated academic debate about the applicability and efficiency of the CRISPR-Cas9 system in editing mtDNA.^36^ Although many studies have confirmed the ability of the CRISPR-Cas9 system to target mitochondria, the efficiency of the mito-Cas9 system in achieving gene knock-in is significantly low. This inefficiency phenomenon is hypothesized to be attributed to two aspects: the lack of mitochondrial translocation efficiency of the editing system, and the limitation of the mtDNA editing ability of Cas9 proteins.^35^ To improve the editing efficiency of the mito-Cas9 system, future research may focus on two directions: first, optimizing the mitochondrial transport mechanism to enhance the efficiency of the editing components entering the mitochondria; second, developing Cas protein variants with higher editing efficiency through genetic engineering.^37^ In summary, through continuous research and exploration, the mito-Cas9 system has the potential to be an efficient knock-in strategy and provide novel therapeutic avenues for the treatment of inherited mitochondrial diseases.

### Mitochondrial base editing system

**Figure 2:**
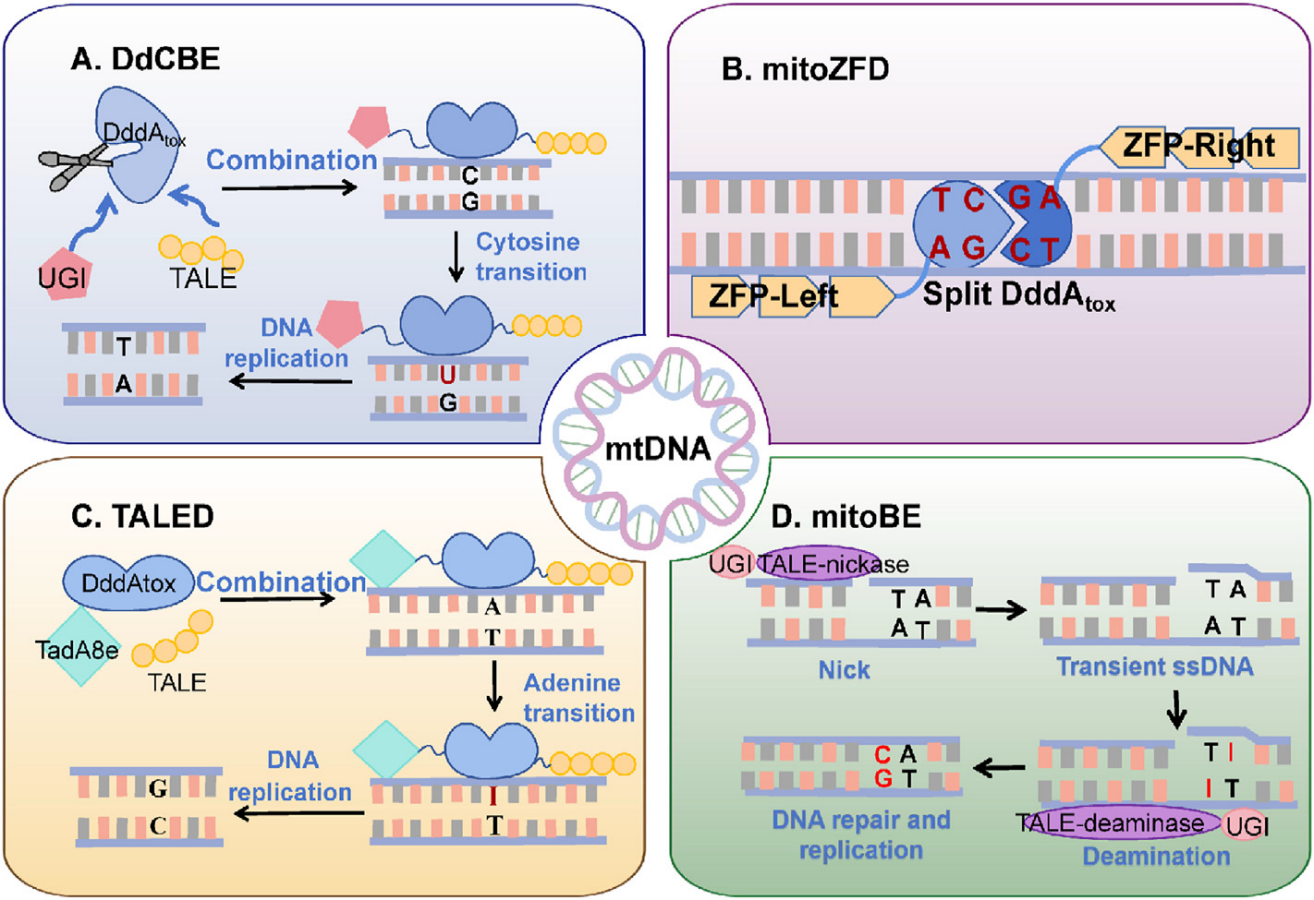
Major mitochondrial base editing tools. **(A)** DdCBEs schematic diagram. DdCBE consists of TALE, half DddAtox, and uracil glycosylase inhibitor (UGI) proteins, which convert C-to-U. U-to-T conversion is then achieved by DNA replication.^40^**(B)** mitoZFD schematic diagram. mitoZFDs consist of zinc-finger DNA-binding proteins, the split interbacterial toxin deaminase DddAtox, and a UGI, which catalyze targeted C-to-T base conversions.^38^**(C)** TALEDs schematic diagram. TALED consists of TALE, TadA8e, and half DddAtox, which converts the A-to-I. I-to-G conversion is then achieved by DNA replication.^41^**(D)** mitoBE schematic diagram. mitoBEs consist of TALE binding proteins with the nickase MutH or Nt. BspD6I(C) and either the single-stranded DNA-specific adenine deaminase TadA8e or the cytosine deaminase ABOBEC1 and UGI, achieving A-to-G or C-to-T base editing.^42^.

Nuclease-mediated gene editing mainly eliminates specific mutant mtDNA, but it cannot correct mutant genomes.^38^ Base editors are double-strand break-free gene editing technologies that open up new possibilities for precise manipulation of mtDNA. This technology can directly substitute individual bases in DNA, mainly cytosine base editors, adenine base editors, and guanine base editors (Fig. 2).^39^ Base editors have demonstrated low off-target and low insertion-deletion rates in nuclear genome engineering applications, resulting in increasing interest in the application of this technology to mitochondrial DNA. We provided a detailed review of the base editors for mitochondrial DNA.

### DdCBE

Mok et al^36^ found that double-stranded DNA deaminase toxin A (DddA) could edit mitochondrial genes by catalyzing the C-to-T transition in dsDNA. To avoid toxicity, DddA is divided into two parts: DddAtox-N and DddAtox-C. TALE was used to identify target sequences, and a uracil glycosylase inhibitor was introduced to improve the editing efficiency (Fig. 2A). The final synthesized protein, named DddA-derived cytosine base editor (DdCBE), was used to model disease-associated mtDNA mutations in human cells, resulting in changes in respiration rates and oxidative phosphorylation.

Compared with nuclease editing systems, DdCBEs have a wide targeting range, high editing efficiency, and high specificity. Silva et al used an adeno-associated virus (AAV) vector to implant DdCBE into the hearts of adult mice via tail vein injection. Editing efficiencies of 2 % and 20 % were detected in mouse heart tissue at weeks 3 and 24 after injection, respectively.^43^ In addition, DdCBE-mediated mitochondrial base editing is also feasible in human 3 PN embryos, suggesting that it can correct pathogenic mtDNA mutations in the early stages of the human embryo.^44^ Recently, Pinheiro et al constructed a base editor library (mitoKO) based on the ability of the DdCBE system to knock out all protein-coding genes in the mouse mitochondrial genome, which will provide a basis for subsequent studies of mitochondrial function and the construction of mtDNA gene inactivation model.^45^

However, when targeting mitochondrial genes in cells, DdBCEs may lead to off-target mutations, which may be due to the spontaneous assembly of DddAtox hemimolecules splitting in the absence of a TALE-DNA interaction.^46^ In addition, the editing efficiency, editing type, and bystander effect of DdCBE still have many shortcomings that need to be improved.^47^ Therefore, researchers have developed many strategies to continuously improve the specificity of DdCBEs and to facilitate their application in basic research and clinical trials of diseases. For example, DdCBEs are fused to nuclear export signals (DdCBE-NES) to avoid off-target C-T transitions in the nuclear genome and to improve mtDNA editing efficiency.^48^ Other optimization strategies, such as modifying the deaminase DddAtox to improve its fidelity, co-expressing repressor proteins of DddA, or modulating the subcellular localization of DdCBEs can also be used to effectively reduce the off-target activity of DdCBEs, laying the groundwork for the application of these tools in gene therapy.^37^^,^^49^

### mitoZFDs

Compared with TALE, ZFNs are small and can be easily packaged in viral vectors for *in vivo* research and gene therapy applications. Simultaneously, ZFPs are somewhat friendly, and the split DddAtox can be fused to either end of the ZFP. In addition, ZFPs with intrinsic cell-penetrating activity may allow nucleic acid-free gene editing in human cells.^50^ Therefore, Kim et al^38^ attempted to fuse DddAtox with specific ZFPs to develop zinc finger deaminases (ZFDs) that catalyze C-to-T base transitions in target DNA and do not induce unwanted insertion and deletion mutations in human cells. The authors successfully improved the gene-editing efficiency of ZFDs by optimizing the linker length, spacer sequence length, and DddAtox splitting site. To deliver ZFDs to the mitochondria, MTS and nuclear export signal sequences were ligated to the N-terminus of ZFDs to form mitoZFDs (Fig. 2B), whose mitochondrial editing efficiencies were in the range of 2.6% to 30% in HEK293T cells. Although ZFDs have achieved relatively high base editing efficiencies in DNA, their relatively low editing efficiencies in mtDNA may limit their application in the treatment of certain mitochondrial diseases. In the future, it is expected that editing efficiency, especially in mtDNA, will be further improved by optimizing design and technology.

### TALEDs

While DdCBEs are limited to C-to-T base editing, Cho et al created a novel gene-editing platform called transcription activator-like effector ligand deaminases (TALEDs), which enabled A-to-G conversion for the first time. TALEDs were created in this study by fusing TALE, TadA8e, and DddAtox, each of which plays a distinct role (Fig. 2C).^51^ TALE is capable of targeting DNA sequences. TadA8e facilitates the A-to-G transition, and DddAtox improves the DNA accessibility of TadA8e. The designed TALED was highly efficient in human cells, catalyzing A-G conversion at 17 target sites across a variety of mitochondrial genes, with an editing frequency of 49%.^40^

TALEDs are non-toxic, have a low off-target risk, largely reduce the bystander effect, and do not lead to mtDNA instability. Furthermore, to reduce off-targeting and improve the efficiency of mitochondrial editing, researchers have further optimized DdCBE and TALED.^52^ The introduction of high-fidelity mutations in DddA and the addition of nuclear export signal sequences to DdCBE and TALED have broad implications for basic research and therapeutic applications.^53^

### mitoBEs

Wei et al^42^ proposed a mitochondrial DNA base editor (mitoBE), in which mitoBEs integrate nickase, deaminases, and uracil glycosylase inhibitors under the guidance of the TALE system. Nickase MutH or Nt.BspD6I(C) cleaves the DNA strand at the target site, generating transient single-stranded DNA, which provides an efficient substrate for deaminases (Fig. 2D). Deaminases, in turn, convert A to G or C to T via deamidation, named mitoABE and mitoCBE, respectively, which enables targeted editing in the human mitochondrial genome, providing a powerful tool for generating mitochondrial disease models or correcting most mitochondrial disease-causing point mutations.

MitoBEs, as a novel mitochondrial single-base editing tool, have lower off-target risks and a higher degree of specificity than DdCBEs. This continues to be explored and optimized in depth. Initially, Wei et al, through mutagenesis studies of nicking enzymes, such as MutH, discovered a substantial expansion of the editable sequence range, achieving a more than 10-fold increase. Furthermore, they investigated more effective delivery methods employing circular RNA-encoded mitoBEs, which enhanced editing efficiency and durability across diverse cell types. Future endeavors may involve the design of an expanded repertoire of nicking enzymes to target various mtDNA sequences or the exploration of novel delivery systems, such as nanoparticles, for the precise localization and enhanced editing efficiency of mitoBEs.

## Delivery systems for mitochondrial gene editing

To function *in vivo*, these gene tools require safe and efficient mitochondria-targeted gene delivery systems that protect them from degradation and overcome extracellular and intracellular barriers, particularly the mitochondrial membrane barrier. Currently, MTS-modified, nanoparticle-based, and viral vector delivery systems are the most common and effective strategies for mitochondrial gene editing.

### MTS-modified delivery systems

The MTS peptide is labeled in the gene editing tool and crosses the mitochondrial bilayer membrane using the mitochondrial protein uptake mechanism, thus facilitating the efficient entry of the editing tool into the mitochondria and realizing the precise editing of mtDNA.^54^ The mitochondrial gene editor composed by MTS has been described in detail above. MTS solves the problem of exogenous proteins, such as zinc finger proteins and TALE, not being able to enter the mitochondria, however, there remains the phenomenon of off-targeting of the nucleus, which needs to be further optimized and researched.^55^ MTS-modified vectors may be cytotoxic, especially as non-specific interactions with the mitochondrial membrane can disrupt mitochondrial membrane integrity. Future designs should optimize the vector or use biocompatible materials to reduce cytotoxicity and non-specific membrane interactions.

### Viral vectors

Viruses, which have evolved to overcome multiple physiological barriers in the body, have become primary vectors for targeting cells using gene-editing drugs. Most gene-editing drugs use AAVs, whereas some use lentiviruses or adenoviruses. Preclinical studies in mice have shown that AAV vectors delivering mitoZFN or mitoTALEN monomers can successfully achieve heterogeneous transfer.^26^^,^^27^ DdCBE targeting the ND4 and ND1 loci was packaged by AAV, and 99.1% and 59.8% efficient editing were detected 6 days after the transfection of HEK293FT cells.^56^ However, AAV vectors also have a limited packaging size of less than 5 kb, and this limits the size and efficient delivery of gene editing tools.^57^ Achieving clinically relevant human doses necessitates impractically high and potentially unsafe titers of AAV vectors, posing a significant challenge for their application in clinical settings. Moreover, when delivering mtDNA editing tools via AAV, considerations include capsid immunogenicity, neutralizing antibodies, and immune responses to AAV-encoded transgenes. Thus, developing novel AAV serotypes with low immunogenicity is crucial for the clinical use of AAV-mediated mtDNA modification tools.^58^

### Non-viral vectors

Non-viral vectors are relatively safe as novel delivery systems with low cytotoxicity and immunogenicity. Lipid nanoparticles are popular non-viral vectors with promising applications in mitochondrial gene therapy; however, they have not yet been used for this purpose. Another type of non-viral vector, virus-like particles, is also showing promise for delivering gene editing tools.^59^ Notably, virus-like particles have a much larger capacity than AAVs (which are limited to 4.3 KB). This means that lipid nanoparticles can carry more complex, diverse, and precise gene-editing tools for the safe delivery of multiple drugs.^58^

## Challenges of mitochondrial gene editing

The development of mitochondrial gene editing tools has enabled the manipulation of mtDNA and the treatment of mitochondrial diseases. However, the mitochondrial bilayer hinders access to exogenous editing tools, and the high variability of mtDNA copy numbers makes precise editing challenging. Improving gene editing tools can significantly enhance the efficiency and precision of mitochondrial gene editing. For example, the TALED editing tool has achieved an editing efficiency of up to 49 %. However, there is still a need to develop safe and controllable vectors for efficient *in vivo* delivery to promote mitochondrial gene therapy.

Off-target effects are important in the clinical application of genome-editing tools. A notable example is the research involving DdCBE. Although DdCBE achieves precise mitochondrial editing, it simultaneously triggers extensive single-nucleotide variant off-target effects on the nuclear DNA.^47^ Given this, editing tools need to be optimized to enhance their base-specific editing ability for mtDNA to improve the precision of mitochondrial gene editing and effectively reduce the risk of off-target editing.

Mitochondrial gene editing enables the treatment of mitochondrial diseases by reducing the intracellular proportion of mutant mtDNA molecules. However, the long-term application of mitochondrial gene editing may affect the dynamics of the cellular proportion of mutant mtDNA molecules, and intracellular mtDNA population dynamics are critical for mitochondrial disease therapy. In addition, long-term mitochondrial editing may lead to undesired mutations in mammalian mitochondria that clonally expand to dangerous levels, thereby affecting mtDNA repair mechanisms.^58^ In this regard, attempts can be made to circumvent this problem using mathematical models to predict the optimal nuclease dose and heterogeneous transfer efficiency.^60^ The long-term impact and safety of these editing technologies must be fully assessed, and comprehensive ethical evaluations must be conducted before they transition from the laboratory to the clinic.

## mtDNA and neurodegenerative disease

The pathogenesis of NDDs is complex and diverse, and our understanding of NDDs is limited to their pathological features. With the development of gene-editing technology, a large number of NDD-associated mutations and single-nucleotide polymorphism loci have been identified, providing new perspectives for explaining the mechanisms of NDD progression. Genetics is a well-recognized determinant of outcome in common NDDs; however, gene mutations do not explain 100 % of NDD cases and the presence of disseminated cases, even in HD, which is usually regarded as an autosomal dominant disorder.^61^

**Figure 3:**
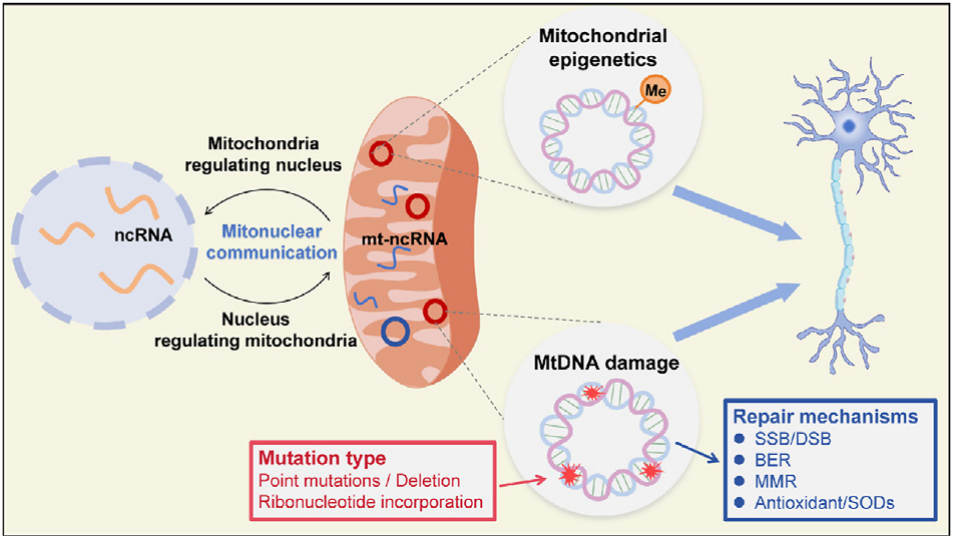
Mitochondrial genetics and epigenetics in neurodegeneration. Mitochondrial dysfunction can arise from mitochondrial genetic or epigenetic alterations. Mitochondrial DNA (mtDNA) is particularly prone to oxidative damage and mutations, including both point mutations and deletions. Moreover, during replication, ribonucleotide analogues (rNMPs) can bind to both nuclear DNA and mtDNA. Repair mechanisms are activated upon mtDNA damage, with the mitochondrial BER pathway being the most extensively studied.^2^ In addition to mtDNA variation, epigenetic mechanisms such as mtDNA methylation and non-coding RNA (ncRNA) play essential roles in mitochondria. These mitochondria-localized ncRNAs, known as nuclear-encoded ncRNAs (nuclear-ncRNAs) or mitochondria-encoded ncRNAs (mt-ncRNAs), are involved in the bidirectional communication between the nucleus and mitochondria, participating in anterograde signaling (nucleus regulating mitochondria) or retrograde signaling (mitochondria regulating nucleus).^65^ BER, base excision repair; DSB, double-stranded break; MMR, DNA mismatch repair; SODs, superoxide dismutases; SSB, single-stranded break.

In the study of NDDs, much less is known about mitochondrial genetics than about their genetics. However, mtDNA genetics have a potential role in the progression of injury-related NDDs (Fig. 3).^62^ Mitochondria are closely associated with oxidative stress and play an important role in maintaining functional homeostasis in the brain and in meeting neuronal energy demands. Much evidence suggests that mitochondria play a central role in NDDs associated with aging and that both mutations in mtDNA and oxidative stress promote aging, which is the greatest risk factor for NDDs.^63^ Mitochondria, like the nucleus, have separate DNA and genes encoding oxidative phosphorylation-related proteins in mtDNA. Mutations in mtDNA occur throughout life due to oxidative damage or errors in mtDNA polymerase, and the clonal expansion of these mutations leads to cellular dysfunction and the production of large amounts of reactive oxygen species, which promotes the development of NDDs.^64^ It has been demonstrated that mtDNA mutations have been shown to play a role in the development of PD and AD. In the following sections, we discuss the effects of mtDNA mutations on NDDs.

### Parkinson's disease

PD is an age-related degenerative disease of motor neurons.^66^ As PD progresses and is prolonged in its course, neuronal loss becomes more extensive and can even lead to dementia and hallucinations.^67^ According to studies, the main pathological factors leading to PD include degenerative death of dopamine neurons in the intracranial substantia nigra,^64^ α-synuclein (α-syn) aggregation, mitochondrial dysfunction, and oxidative stress.^68^ In addition, mtDNA alterations play a key role in the progression of PD.^69^^,^^70^ Currently, the main types of mtDNA mutations that may be associated with PD include point mutations, deletions, and regulation of nuclear genes.^71^^,^^72^

It was shown that the age-related increase in oxidative mtDNA damage is associated with age-related impairments of the components of the mitochondrial BER machinery (OGG1, UDG, APE1, and polymerase γ).^73^ Furthermore, studies have shown that the dysregulation of mtDNA homeostasis is a key process in the pathogenesis of neuronal loss in PD.^74^ For example, Christian et al found that mtDNA deletions and copy number regulation, rather than point mutations, were the main determinants of mtDNA damage in human neurons. It was also found that in the context of PD, the dynamic regulation of mtDNA copy number is weakened, leading to the depletion of the wild-type mtDNA library, which may contribute to mitochondrial respiratory defects in neurons.^75^ Another study found that multiple mtDNA deletions accumulated in the substantia nigra became more pronounced with age. This has been associated with nuclear genes encoding proteins associated with mitochondrial maintenance, such as the gene encoding Pol-γ, POLG.^76^ Other nuclear genes associated with impaired mtDNA maintenance, such as c10orf2 and MPV171, have also been detected in other studies of PD.^77^^,^^78^ Mitochondrial dysfunction can also be caused by mitochondrial epigenetic changes. A recent review highlighted that mitochondrial epigenetics might play an important role in the development and progression of PD and should receive more attention. Mechanistic modulation of the epigenome also appears to be critical for the pathogenesis and progression of PD.^48^ Blanch et al detected methylation deletions at mtDNA CpG and non-CpG sites in the nigral striatal D-loop region of PD patients. Methylation may regulate mtDNA replication and may be associated with mtDNA deletion as well.^79^

Several studies have developed gene therapy approaches specifically for PD.^80^, ^81^, ^82^ However, none of these approaches have targeted the underlying pathophysiological features of PD. This is because the unique features of the nuclear and mitochondrial genomes, and the interaction between the two have been implicated in the pathogenesis of PD. A combination of different gene therapies may be the most effective treatment strategy.^83^ We suggest that targeted mitochondrial gene therapy may offer opportunities for treating PD and advance the field of PD gene therapy.

### Alzheimer's disease

AD is a common neurodegenerative disorder. It is caused by the loss of neurons in the cerebral cortex and hippocampus, aggregation of β-amyloid (Aβ), and neurofibrillary tangles, leading to neuronal degeneration and death.^84^ Oxidative stress associated with mitochondrial dysfunction is another potential factor contributing to the pathogenesis of AD by inducing the conformational dysregulation of proteins, particularly α-synaptic nucleoprotein, Aβ, and tau.^85^ Several studies have shown that mitochondrial DNA changes play a key role in the pathogenesis of AD. Currently, the main types of mtDNA mutations associated with AD include deletions, point mutations, and methylation.

mtDNA mutations play a crucial role in AD-related mitochondrial dysfunction. Using ultrasensitive next-generation sequencing to measure the mutational load of the mitochondrial genome, results have shown a significant increase in the frequency of mtDNA mutations in the hippocampus of individuals with early AD.^86^ In addition, Krishnan et al found that high levels of mtDNA deletion led to COX deficiency, and they observed an increase in mitochondrially biochemically deficient neurons in the hippocampus of sporadic AD patients with COX deficiency.^87^^,^^88^ Further studies have shown an association between the T→C transition in the gene encoding the COX III subunit and reduced citrate synthase activity, with up to 15% of AD patients exhibiting this specific mtDNA point mutation.^58^ Recent studies have successfully detected mitochondrial epigenetic alterations in the central nervous system, cerebrospinal fluid, and periphery.^89^ In particular, mtDNA methylation emerges as one of the key factors that may regulate mitochondrial pathology in AD. Blanch et al found increased mtDNA methylation at both CpG and non-CpG sites within the D-loop region of the inner olfactory cortex in AD patients with Braak stages I-II and III-IV versus controls.^63^ In another study, the D-loop region was demethylated and the mtDNA copy number was reduced in APP/PS1 mice, an animal model of familial AD.^90^ Collectively, these findings suggest that mtDNA has significant potential as a biomarker and therapeutic target for AD.

Although mtDNA plays a key role in the pathogenesis of AD, there are no relevant studies on targeting mitochondrial genes for treating AD. Currently, CRISPR/Cas9 technology can target the editing of genes associated with AD pathogenesis, such as APP, PSEN1, and APOE, to reduce the production of harmful proteins or increase the expression of protective proteins.^91^^,^^92^ The combination of CRISPR/Cas9 gene editing tools with targeted mitochondrial gene editing technology holds great promise, as it may allow for a more comprehensive approach to treating AD by addressing both nuclear and mitochondrial genetic factors that contribute to the disease. While challenges such as efficient delivery and minimizing off-target effects remain, future research efforts focused on the integration of these technologies hold the key to unlocking new avenues for the prevention and treatment of AD.

### Huntington's disease

**Figure 4:**
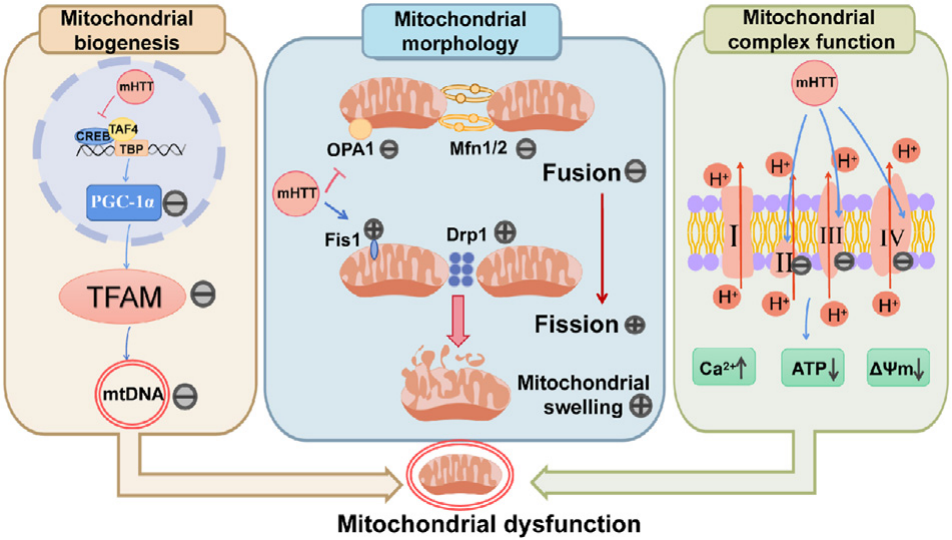
Differential effects of mHTT on mitochondria in Huntington's disease. (i) Effects on mitochondrial biosynthesis: mHTT interferes with the PGC-1α transcriptional pathway, leading to reduced TFAM activation and, consequently, disruption of mitochondrial biosynthesis. (ii) Effects on mitochondrial morphology: mHTT expression increases the levels of Drp1 and Fis1 while decreasing the levels of Mfn1/2 and OPA1. This disrupts the balance between mitochondrial fusion and division, ultimately resulting in increased mitochondrial swelling. (iii) Effect on respiratory chain function: mHTT expression leads to a decrease in the function of respiratory chain complexes II, III, and IV. This, in turn, results in decreased ATP production, calcium overload, and a decrease in membrane potential.^96^ Drp1, dynamin-related protein 1; Fis1, fission 1; Mfn1/2, mitofusin 1/2; mHTT, mutant Huntington's protein; mtDNA, mitochondrial DNA; Opa1, optic atrophy 1; PGC-1α, peroxisome proliferator-activated receptor-gamma coactivator-1alpha; TFAM, mitochondrial transcription factor A.

HD is a degenerative disease of the central nervous system, characterized by cognitive decline, psychiatric disorders, and motor dysfunction. Its main pathological manifestation is the significant absence of spiny projection neurons in the striatum, a crucial component of the extrapyramidal system. This neuronal loss, being extensive, results in impaired limb motor function.^93^ HD is an autosomal dominant disorder primarily caused by the amplification of the CAG trinucleotide repeat sequence within the Huntington's disease gene (HTT), ultimately leading to the production of mutant Huntington's protein (mHTT), which structurally differs from its normal counterpart.^94^ Mitochondrial dysfunction has been found in the brain and peripheral tissues of patients diagnosed with HD. Several studies have suggested that impaired mitochondrial function and excitatory neurotoxicity may be the intracellular molecular mechanisms by which mHTT induces the early onset of HD (Fig. 4).^95^

Notably, mitochondrial function is a complex entity that is not only dictated by nuclear DNA but also crucially influenced by mtDNA. Wang et al investigated mtDNA heterogeneity in lymphoblastoid and longitudinal blood samples from HD patients, discovering that higher levels of pathogenic heterogeneity correlated with reduced functional capacity and deterioration of motor and cognitive functions, highlighting its importance in HD progression. Furthermore, the study revealed that the occurrence of pathogenic heterogeneity in HD samples spanned all 13 protein-coding genes in the mtDNA.^97^ Mutations and deletions in the mtDNA, as well as reduced activity of mitochondrial complexes II and III, have been observed in HD mouse models and patients.^98^ Specifically, mtDNA alterations have been demonstrated in both the R6/2 mouse^99^ and the STHdhQ111 mouse^100^ models of HD, with the former showing reduced mtDNA levels in the striatum and the latter showing increased mtDNA damage.^58^ mtDNA mutation-induced mitochondrial dysfunction affects the pathogenesis of HD, but whether defective mtDNA accumulation occurs in the early stages of HD and whether it can be detected *in vivo* is controversial.

HD primarily relies on pharmacological interventions. In the mHTT mouse model, a series of antioxidants have been used to target mouse mitochondria, resulting in the restoration of mitochondrial function, reduction of reactive oxygen species levels, and improvement of the HD phenotype.^78^ Given the monogenic nature of HD, gene therapy holds promise for targeting the affected nucleus.^79^ Although direct gene therapy targeting mitochondria remains an area of ongoing research, it is noteworthy that the m.3244 mutation (*n* = 21), located within the tRNALeu gene, is highly prevalent in HD samples.^81^ Studies have demonstrated that the use of mitoTALENs in the tRNA^Ala^ m.*5024C* > T mutant mouse model can significantly decrease the levels of mutant mtDNA.^31^ Envisioning the future, the development of mitochondrial base editors tailored to the tRNA^Leu^ locus could potentially mitigate or delay the progression of HD.

### Amyotrophic lateral sclerosis

Amyotrophic lateral sclerosis (ALS) is the most common motor neuron disease characterized by the progressive degeneration of the upper and lower motor neurons.^101^ The pathogenesis is complex and includes excitotoxicity, accumulation of TDP-43 and proteins, neuroinflammation, mitochondrial dysfunction, and oxidative stress. While much of the previous research has centered on the epigenetics of nuclear genes, it is now recognized that mitochondrial genomic epigenetics also significantly contributes to the development of ALS. The DNA methylation level in the regulatory region (D-loop) of mitochondrial DNA is dynamically disturbed during the progression of ALS, while the D-loop plays a critical role in regulating the replication and transcription of mitochondrial DNA.^102^ One study found that in ALS patients carrying SOD1 or C9orf72 gene mutations, despite an increase in the copy number of mtDNA, the methylation level of the D-loop showed a downward trend. More strikingly, there is a significant inverse correlation between the methylation level of the D-loop and the copy number of mtDNA. This discovery not only reveals the close relationship between the two but also suggests the possible existence of a compensatory mechanism.^86^

Clinical trials of gene therapy for ALS patients with SOD1 mutations, C9orf72 hexanucleotide repeat expansions, ATXN2 trinucleotide expansions, and FUS mutations are underway.^103^ However, many studies on ALS have shown that mtDNA damage is closely associated with mutations in proteins encoded by nuclear genes, and the use of targeted mitochondrial gene editing tools to regulate the balance between healthy and mutated mtDNA in future gene therapies for ALS may further advance the development of gene therapy for ALS.

## Conclusion and outlook

As societies age, the prevalence of individuals with NDDs is gradually rising. This trend is gradually shifting towards younger individuals, thereby creating a need for more therapeutic approaches and strategies for these diseases. Many studies have shown that a close correlation between mitochondrial DNA (mtDNA) mutations and NDDs. The potential restoration of normal mitochondrial function through repairing damaged mtDNA genes holds promise for mitigating the progression of these disorders. This review aims to summarize and discuss the association between mtDNA mutations and four common NDDs (PD, AD, HD, and ALS). Furthermore, it delves into the underlying pathogenesis of mtDNA mutations in these conditions.

In recent years, there have been impressive developments in gene-editing technology, from artificial nuclease editing systems to mitochondrial base editing systems. These breakthroughs continue to improve our ability to manipulate mtDNA and provide more effective techniques for mtDNA editing. mtDNA editing technologies can eliminate or correct mutated mtDNA, reducing the proportion of mtDNA below the disease threshold and restoring the clinical phenotype. Continued optimization of existing editing technologies and different gene therapy approaches may potentially rescue mtDNA dysfunction in NDDs and slow down the disease process.

## Conflict of interests

The authors declared no competing interests.

## Funding

This study was supported by grants from the 10.13039/501100004731Zhejiang Provincial Natural Science Foundation of China (No. LD22H090002), the 10.13039/501100001809National Natural Science Foundation of China (No. 82174038), and the horizontal scientific research project of 10.13039/501100008327Zhejiang University of Technology (China) (No. KYY-HX-20180348).

## CRediT authorship contribution statement

**Ye Hong:** Writing – original draft. **Ying Song:** Writing – review & editing. **Wenjun Wang:** Writing – review & editing. **Jinghui Shi:** Investigation. **Xi Chen:** Writing – review & editing.
